# Prevalence of Overweight/Obesity and Its Associated Factors among University Students from 22 Countries

**DOI:** 10.3390/ijerph110707425

**Published:** 2014-07-21

**Authors:** Karl Peltzer, Supa Pengpid, T. Alafia Samuels, Neslihan Keser Özcan, Carolina Mantilla, Onja H. Rahamefy, Mee Lian Wong, Alexander Gasparishvili

**Affiliations:** 1 ASEAN Institute for Health Development, Madidol University, Salaya, Phutthamonthon, Nakhonpathom 73170, Thailand; 2 Department of Psychology, University of the Free State, Bloemfontein 9300, South Africa; 3 HIV/AIDS/STIs and TB (HAST) Research Programme, Human Sciences Research Council, Private Bag X41, Pretoria 0001, South Africa; 4 Department of Research, University of Limpopo, Turfloop Campus, Sovenga 0727, South Africa; E-Mail: supaprom@yahoo.com; 5 Faculty of Medical Sciences, University of the West Indies, Cave Hill Campus, P.O. Box 64, Bridgetown 11000, Barbados; E-Mail: alafiasam@gmail.com; 6 Department of Midwifery, Faculty of Health Science, Istanbul University, Bakirkoy, Istanbul 34740, Turkey; E-Mail: neslihan_keser@hotmail.com; 7 Departamento de Fisioterapia, Universidad de Pamplona, Pamplona 0, Colombia; E-Mail: sonia.mantilla@unipamplona.edu.co; 8 Research and Training Laboratory of Medical Biology, Faculty of Medicine, University of Antananarivo, Antananarivo 001, Madagascar; E-Mail: onja_holisoa@yahoo.com; 9 School of Public Health, National University of Singapore, 16, Medical Drive, Singapore 117597, Singapore; E-Mail: mee_lian_wong@nuhs.edu.sg; 10 Centre for Sociological Studies, Moscow State University, Moscow 119991, Russia; E-Mail: gasparishvili@yandex.ru

**Keywords:** overweight, obesity, body mass index, dietary pattern, social determinants, health behaviour, mental health, childhood abuse, university students, 22 countries

## Abstract

Obesity among young people increases lifetime cardiovascular risk. This study assesses the prevalence of overweight/obesity and its associated factors among a random sample of university students from 22 universities in 22 low, middle income and emerging economy countries. This cross-sectional survey comprised of a self-administered questionnaire and collected anthropometric measurements. The study population was 6773 (43.2%) males and 8913 (56.8%) females, aged 16 to 30 years (mean 20.8 years, SD = 2.6). Body mass index (BMI) was used for weight status. Among men, the prevalence of underweight was 10.8%, normal weight 64.4%, overweight 18.9% and obesity 5.8%, while among women, the prevalence of underweight was 17.6%, normal weight 62.1%, overweight 14.1% and obesity 5.2%. Overall, 22% were overweight or obese (24.7% men and 19.3% women). In multivariate regression among men, younger age, coming from a higher income country, consciously avoiding fat and cholesterol, physically inactivity, current tobacco use and childhood physical abuse, and among women older age, coming from a higher income country, frequent organized religious activity, avoiding fat and cholesterol, posttraumatic stress symptoms and physical childhood abuse were associated overweight or obesity. Several gender specific risk factors identified can be utilized in health promotion programmes.

## 1. Introduction

Globally, there is rising prevalence of overweight and obesity in both developing and developed countries [1]. The rate of obesity has tripled in developing countries over the past 20 years as they rapidly become more urbanized, with increased consumption of high calorie foods and adoption of a more sedentary lifestyle [1,2]. Some studies observed that first year university students have significant weight gain [3], followed by ongoing slow but steady increase in weight [4]. 

Studies among university students in developing countries show high prevalence of overweight and obesity: Africa (Nigeria: 10% [5]; Egypt: 25.3%–59.4% [6,7], South Africa: 10.8%–24% [8,9]; Asia (Bangladesh: 20.8% [10]; China: 2.9%–14.3% [11,12]; Malaysia: 20%–30.1% [13,14], Thailand: 31% [15], Pakistan: 13%–52.6% [16,17], and India: 11%–37.5% [18,19,20,21]; Latin America (Colombia: 12.4%–16.7% [22]; Mexico: 31.6% [23], the Middle and Near East (Saudi females: 47.9% [24], Oman: 28.2% [25] Kuwait: 42% [26,27], Iran 12.4% [28], and Turkey: 10%–47.4% [29,30]). Although there are prior studies on obesity/overweight in students in individual developing countries, this study collected data from 22 countries (low, lower middle, upper middle and high income countries) in different regions (Africa, Asia, The Caribbean and South America) using the same methodology, which allows for direct comparison across countries.

In a previous review [21], the following factors were identified to be associated with overweight and obesity among university students or (young) adults: (1) socio-demographic factors (mainly male gender, older age and higher socioeconomic status; (2) Social factors: lack of social support, capital and lack of religiousness; (3) Dietary behaviour: intakes of fiber, consumption of red meat, skip breakfast more often, high number of meals, snacking behaviour; and (4) Health risk behaviour: Physical inactivity, frequent alcohol use, and smoking; (5) Mental health and childhood abuse: poor mental health (depression, anxiety) and childhood physical abuse, sexual and verbal abuse.

The purpose of this study was to assess the prevalence of overweight/obesity and its associated factors among university students in 22 low and middle income and emerging economy countries.

## 2. Methods

### 2.1. Sample and Procedure

This cross-sectional study was carried out with a network of collaborators in participating countries (see Acknowledgments). The anonymous, self-administered questionnaire was developed in English, translated into the Arabic, Bahasa, French, Lao, Russian, Spanish, Thai and Turkish languages of the participating countries, then back-translated to English. In each country where translated questionnaires were used, they were pilot tested for face validity and understanding with 25 students who were not from the sample population. The study was initiated through personal academic contacts of the principal investigators. In 2013, these collaborators arranged for data to be collected by trained research assistants from intended 400 male and 400 female undergraduate university students aged 16–30 years in one or two universities located in the capital or other major cities. Classes of students from all years of study and across multiple faculties were recruited by timetable scheduling using stratified random sampling. Research assistants asked these classes of undergraduate students to complete the questionnaire at the end of a teaching session, followed by the anthropometric measurements. We included no incentive for participation, and there were no penalties for refusal. Written informed consent was obtained from participating students. The required ethics approvals were obtained by all participating institutions. 

### 2.2. Measures

#### 2.2.1. Anthropometric Measurements

Students were weighed and measured by trained researchers using standardised protocols [31]. Standing height was measured to the nearest 0.1 cm without shoes, using a stadiometer. Participants wearing light clothes, were weighed to the nearest 0.01 kg, on a load-cell-operated digital scale which was first calibrated using a standard weight and re-checked daily [32]. Body mass index (BMI) was calculated as weight in kg divided by height in metres squared. In South and East Asian participants overweight and obesity were defined as ≥23.0–27.4 kg/m^2^ and ≥27.5 kg/m^2^ respectively [33] and for the other countries ≥25.0–29.9 kg/m^2^ and ≥30 kg/m^2^, respectively [34].

Socio-demographic questions included age, gender, and marital status. Socioeconomic background was assessed by relative family income within each country as wealthy (within the highest 25%), quite well off (within the 50% to 75% range), not very well off (within the 25% to 50% range), or quite poor (within the lowest 25%) [35]. 

#### 2.2.2. Social Variables

Three items from the Social Support Questionnaire were utilized to assess perceived social support [36]. The questions were selected, as in previous research [37], to assess perceived tangible and emotional support: “If I were sick and needed someone to take me to a doctor I would have trouble finding someone (reversed); I feel that there is no one I can share my most private concerns and fears (reversed); and I feel a strong emotional bond with at least one other person”. Cronbach alpha for this social support index was 0.95. *Organized religious activity* was assessed with one item from the Duke University Religion Index (DUREL) [38].

#### 2.2.3. Dietary Behaviour Variables

Fruit and vegetable (FV) consumption was assessed with two questions “How many servings of fruit do you eat on a typical day?” and “How many servings of vegetables do you eat on a typical day?” using the 24-h dietary recall data as the gold standard [39]. Cronbach alpha for this fruit and vegetable measure was 0.74. Insufficient FV consumption was defined as less than five servings of fruits and/or vegetables a day [39]. Additional dietary variables included: (a) frequency of consumption of red meat (daily, 2–3 times a week, once a week, less than once a week, never); (b) trying to avoid eating foods that contain fat and cholesterol (yes, no); (d) trying to eat foods that are high in fibre (yes, no); (e) frequency of having breakfast; (f) frequency of between-meal snacks and (g) number of meals a day [40].

#### 2.2.4. Health Risk Behaviour

*Physical activity* was assessed using the self-administered International Physical Activity Questionnaire (IPAQ) short version, for the last 7 days (IPAQ-S7S). We used the instructions given in the IPAQ manual for reliability and validity, which is detailed elsewhere [41]. We categorized physical activity (short form) according to the official IPAQ scoring protocol [42] as low, moderate and high. 

*Tobacco use* was assessed with the question: Do you currently use one or more of the following tobacco products (cigarettes, snuff, chewing tobacco, cigars, *etc.*)? Response options were “yes” or “no” [43]. *Heavy alcohol* consumption was measured by asking participants “how often do you have (for men) five or more and (for women) four or more drinks on one occasion?” 

#### 2.2.5. Mental Health and Abuse

We assessed depressive symptoms using the 10-item version of the Centres for Epidemiologic Studies Depression Scale (CES-D)*.* Scores of 0–9, 10–14 and 15 or more indicative of mild, moderate and severe depressive [44]. The Cronbach alpha reliability coefficient of this 10-item scale was 0.74 in this study.

Breslau’s 7-item screener was used to identify Post traumatic stress disorder (PTSD) symptoms in the past month [45]. Items asked whether the respondent had experienced difficulties related to a traumatic experience (e.g., “Did you begin to feel more isolated and distant from other people?”). Participants who answered affirmatively to at least four of the questions were considered to have a positive screen for PTSD [45]. The Cronbach alpha reliability coefficient of this 7-item scale was 0.75 in this study.

*Child abuse* was assessed with two questions, regarding being physically abused as a child and being sexually abused as a child. 

### 2.3. Data Analysis

The data were analysed using IBM SPSS Statistics for Windows (Version 20.0., Armonk, NY, USA). Sociodemographic factors, social and dietary variables, health risk behaviours, mental health, childhood abuse and BMI categories were calculated as percentages. Multivariate logistic regression was performed for men and women separately with overweight/obesity as the dependent variable. Potential multi-collinearity between variables was assessed with variance inflation factors, none of which exceeded the recommended critical value of 4.0 [46]. Socio-demographic characteristics, social, health risk behaviours, mental health and childhood abuse variables were taken as independent variables. *p* < 0.05 was considered significant. The country was entered as the primary sampling unit for survey analysis in STATA in order to achieve accurate CIs, given the clustered nature of the data.

## 3. Results and Discussion

### 3.1. Sample Characteristics

The total sample included 15,746 undergraduate university students (mean age 20.8, SD 2.6, age range of 16–30 years) from 22 countries. The students were from faculties of education, humanities and arts, social sciences, business and law, science, engineering, manufacturing and construction, agriculture, health and welfare and services. Participation rate in most countries was over 90%.

Table 1 and Table 2 show the numbers of male and female university student participants by country, the mean age, measured height and weight, calculated BMI, and the proportion of students classified as underweight, normal weight, overweight and obese. Among men, the prevalence of underweight was 10.8%, normal weight 64.4%, overweight 18.9% and obesity 5.8%, while among women, the prevalence of underweight was 17.6%, normal weight 62.1%, overweight 15.1% and obesity 5.2%. Table 3 shows the prevalence of overweight/obesity by gender and socio-economic profile.

**Table 1:** Reported mean age, measured height and weight, and calculated BMI and weight status among male university students from 22 low and middle income and emerging economy countries, 2013.

Country	N	Age (years) (SD)	Height (m) (SD)	Weight (kg) (SD)	BMI	Under Weight	Normal Weight	Over Weight	Obesity
**All**	6773	21.0 (2.6)	1.73 (0.09)	67.3 (13.2)	22.5 (4.1)	10.8	64.4	18.9	5.8
**Carribbean and Latin America**									
Barbados ^4^	330	21.6 (2.6)	1.76 (0.08)	77.2 (17.5)	24.9 (5.0)	6.1	54.5	26.4	13.0
Jamaica ^3^	158	20.9 (4.2)	1.76 (0.12)	73.0 (15.6)	23.4 (4.2)	3.8	69.0	21.5	5.7
Colombia ^3^	357	21.4 (3.7)	1.72 (0.06)	69.3 (12.1)	23.5 (3.6)	4.5	66.1	24.4	5.0
Venezuela ^3^	173	21.0 (2.7)	1.74 (0.15)	71.3 (13.4)	23.3 (3.0)	4.0	71.7	19.1	5.2
**Sub-Saharan Africa**									
Ivory Coast ^2^	396	24.0 (2.5)	1.72 (0.10)	63.0 (8.1)	21.3 (2.8)	7.8	85.4	6.1	0.8
Madagascar ^1^	387	20.3 (1.8)	1.68 (0.06)	58.7 (7.3)	20.7 (2.3)	16.0	79.3	4.4	0.3
Mauritius ^3^	142	21.4 (1.1)	1.75 (0.08)	66.8 (14.0)	21.7 (4.1)	20.4	62.0	15.5	2.1
Namibia ^3^	111	20.6 (1.1)	1.73 (0.06)	66.1 (11.7)	22.0 (3.9)	10.8	72.1	11.7	5.4
Nigeria ^2^	439	21.1 (2.6)	1.73 (0.11)	65.2 (12.1)	21.4 (2.8)	11.8	77.4	10.0	0.7
South Africa ^3^	315	22.3 (3.4)	1.67 (0.20)	64.7 (10.6)	22.3 (3.5)	9.8	73.0	13.3	3.8
**North Africa, Near East and central Asia**									
Egypt ^2^	321	20.8 (1.3)	1.75 (0.08)	78.0 (13.9)	25.4 (4.5)	2.8	46.4	38.3	12.5
Tunesia ^3^	295	23.7 (4.1)	1.78 (0.07)	74.8 (13.7)	23.7 (4.1)	4.1	67.1	23.4	5.4
Turkey ^3^	398	20.7 (2.3)	1.77 (0.07)	73.4 (11.6)	23.4 (3.4)	3.0	73.1	19.6	4.3
Russia ^3^	381	20.1 (1.9)	1.79 (0.09)	76.7 (11.0)	23.7 (3.3)	2.9	69.0	24.4	3.7
Kyrgyzstan ^1^	349	21.3 (1.5)	1.75 (0.06)	67.6 (9.2)	22.0 (2.7)	6.6	82.5	9.2	1.7
**South Asia**									
Bangladesh ^1^	358	21.6 (2.1)	1.69 (0.08)	67.8 (13.6)	23.8 (4.9)	6.1	47.2	30.4	16.2
India ^2^	541	17.9 (0.6)	1.67 (0.09)	63.2 (11.9)	22.7 (4.5)	11.8	47.9	28.5	11.8
Pakistan ^2^	319	20.2 (1.8)	1.77 (0.11)	57.2 (5.1)	18.1 (1.9)	61.1	36.7	2.2	0.0
**Southeast Asia**									
Laos ^2^	260	22.6 (1.8)	1.63 (0.07)	56.7 (7.8)	21.3 (2.8)	8.1	68.1	19.2	4.6
Philippines ^2^	196	18.4 (1.5)	1.68 (0.22)	60.9 (14.4)	21.9 (5.0)	19.9	46.4	22.4	11.2
Singapore ^4^	328	21.9 (1.6)	1.74 (0.07)	66.4 (11.8)	22.1 (4.0)	8.8	61.9	23.8	5.5
Thailand ^3^	219	20.4 (1.3)	1.72 (0.07)	64.5 (11.9)	21.9 (3.8)	13.7	58.0	19.6	8.7

Notes: **^1^** Low income country; **^2^** Lower middle income country; **^3^** Upper middle income country; **^4^** High income country (Source: World Bank, New Country Classifications, 2013 [47]).

**Table 2:** Reported mean age, measured height and weight, and calculated BMI and weight status among female university students from 22 low and middle income and emerging economy countries, 2013.

Country	N	Age (years) (SD)	Height (m) (SD)	Weight (kg) (SD)	BMI	Under Weight	Normal Weight	Over Weight	Obesity
**All**	8913	20.7 (2.6)	1.61 (0.09)	56.9 (11.8)	21.9 (4.2)	17.6	62.1	15.1	5.2
**Carribbean and Latin America**									
Barbados ^4^	246	21.6 (3.0)	1.64 (0.07)	68.4 (21.3)	25.5 (7.7)	11.8	47.6	22.0	18.7
Jamaica ^3^	516	21.1 (4.9)	1.64 (0.09)	63.1 (15.3)	23.4 (5.6)	11.8	60.1	17.4	10.7
Colombia ^3^	453	20.8 (2.8)	1.59 (0.06)	58.3 (9.2)	23.0 (3.8)	6.2	71.5	16.8	5.5
Venezuela ^3^	271	20.5 (2.8)	1.62 (0.07)	57.6 (10.1)	22.0 (3.6)	13.7	68.3	13.7	4.4
**Sub-Saharan Africa**									
Ivory Coast ^2^	381	23.5 (2.8)	1.63 (0.06)	58.3 (9.2)	21.9 (3.2)	12.9	70.3	14.7	2.1
Madagascar ^1^	393	19.6 (1.5)	1.56 (0.06)	50.7 (7.7)	20.8 (2.8)	20.1	74.3	4.6	1.0
Mauritius ^3^	319	20.7 (1.2)	1.60 (0.07)	52.3 (12.6)	20.3 (4.6)	37.6	51.4	6.6	4.4
Namibia ^3^	317	22.1 (4.1)	1.61 (0.12)	58.9 (12.1)	22.7 (5.7)	16.7	61.8	14.5	6.9
Nigeria ^2^	361	21.4 (2.7)	1.62 (0.17)	60.0 (16.4)	21.7 (3.6)	17.7	65.9	13.3	3.0
South Africa ^3^	430	22.2 (22.2)	1.57 (0.13)	62.9 (13.5)	25.1 (5.2)	4.0	55.1	25.3	15.6
**North Africa, Near East and central Asia**									
Egypt ^2^	375	20.3 (1.3)	1.61 (0.11)	63.2 (11.7)	24.0 (4.0)	4.8	61.3	25.3	8.5
Tunesia ^3^	615	21.0 (1.7)	1.65 (0.06)	62.5 (9.9)	23.0 (3.6)	7.3	67.3	20.3	5.0
Turkey ^3^	397	20.5 (2.1)	1.64 (0.06)	58.0 (9.5)	21.5 (3.1)	16.1	70.3	11.8	1.8
Russia ^3^	404	19.8 (1.7)	1.67 (0.06)	57.3 (8.6)	20.5 (2.9)	24.0	69.3	5.9	0.7
Kyrgyzstan ^1^	465	21.3 (1.4)	1.64 (0.06)	55.7 (8.3)	20.7 (2.9)	24.1	68.0	7.3	0.6
**South Asia**									
Bangladesh ^1^	283	20.5 (1.8)	1.63 (0.08)	56.3 (10.4)	21.2 (3.3)	18.4	63.3	13.1	5.3
India ^2^	259	18.0 (0.9)	1.61 (0.08)	56.6 (11.2)	21.8 (4.1)	13.5	56.8	22.0	7.7
Pakistan ^2^	442	19.6 (1.9)	1.58 (0.06)	53.1 (5.8)	21.3 (2.6)	14.5	61.3	23.3	0.9
**Southeast Asia**									
Laos ^2^	499	22.1 (1.9)	1.56 (0.06)	50.5 (8.8)	20.8 (3.6)	28.7	52.5	14.6	4.2
Philippines ^2^	573	18.3 (1.3)	1.54 (0.09)	49.3 (10.6)	20.6 (3.6)	27.1	54.3	14.1	4.5
Singapore ^4^	348	20.5 (1.4)	1.61 (0.06)	52.3 (8.7)	20.2 (3.0)	30.2	54.3	13.2	2.3
Thailand ^3^	566	20.0 (1.2)	1.61 (0.05)	53.6 (9.7)	20.8 (3.6)	24.6	57.6	12.4	5.5

Notes: **^1^** Low income country; **^2^** Lower middle income country; **^3^** Upper middle income country; **^4^** High income country (Source: World Bank, New Country Classifications, 2013 [47]).

**Table 3:** Prevalence characteristics of overweight/obesity participants by gender among university students from 22 low and middle income and emerging economy countries, 2013.

Variable	All	Men	Women	Statistic
	N (%) or M (SD)	N (%) or M (SD)	N (%) or M (SD)	*p*-value
**Socio-Demographics**				
*All*	3495 (22.0)	1676 (24.7)	1812 (20.3)	
*Age in years*				<0.001
16–19	1078 (21.8)	513 (26.3)	565 (18.9)	
20–21	1091 (19.7)	494 (21.6)	597 (18.4)	0.004
22 or more	1186 (25.6)	616 (26.7)	566 (24.5)	
Wealth				
Wealthy	184 (25.9)	116 (31.9)	68 (19.7)	
Quite well off	1699 (22.2)	813 (25.8)	882 (19.7)	<0.001
Not well off	1309 (22.2)	576 (23.5)	730 (21.4)	
Poor	272 (21.2)	152 (21.8)	120 (20.7)	
Country income classification				
Low income	337 (15.0)	223 (20.4)	111 (9.7)	
Lower middle income	1225 (22.8)	590 (23.9)	635 (22.8)	<0.001
Upper middle income	1311 (22.0)	552 (24.5)	756 (20.6)	
High income	231 (40.0)	130 (39.4)	100 (40.7)	
**Social variables**				
Organized religious activity (Range 1–6)	3.9 (1.6)	3.8 (1.7)	3.9 (1.6)	0.815
Social support (Range 3–12)	9.1 (2.0)	9.0 (2.1)	9.3 (1.9)	<0.001
**Dietary variables**				
Eats red meat at least once a day	1527 (22.5)	720 (25.0)	806 (20.8)	<0.001
Try to eat fiber	1414 (24.1)	583 (26.6)	829 (22.7)	<0.001
Avoids fat and cholesterol	1496 (25.2)	631 (28.2)	863 (23.4)	<0.001
Fruit and vegetables (<5 times/day)	2500 (21.9)	1209 (24.7)	1289 (19.8)	<0.001
Skipping breakfast	1617 (22.3)	737 (24.3)	878 (20.9)	0.013
Number of meals a day	2.7 (0.8)	2.7 (0.8)	2.6 (0.7)	<0.001
Number of in-between snacks	1.5 (0.9)	1.5 (0.9)	1.6 (0.9)	<0.001
**Health risk behaviour**				
Physical activity				
Low	1703 (22.7)	706 (26.8)	994 (20.7)	
Moderate	700 (21.0)	326 (24.4)	370 (18.7)	<0.001
High	1092 (22.2)	644 (23.0)	448 (21.1)	
Current tobacco use	517 (27.1)	408 (29.8)	109 (20.5)	<0.001
Binge drinking (past month)	508 (24.3)	327 (26.4)	181 (21.4)	<0.001
**Mental health and abuse**				
Depression (severe)	552 (25.8)	249 (29.1)	303 (23.7)	0.094
PTSD	746 (23.9)	333 (26.1)	413 (22.6)	0.010
Child sexual abuse	114 (28.1)	46 (32.2)	68 (26.1)	0.179
Child physical abuse	216 (28.7)	104 (28.5)	112 (29.2)	0.568

### 3.2. Prevalence of Overweight/Obesity

Overall, 22% were overweight or obese; men (24.7%) were significantly more overweight or obese than women (20.0%) (see Table 3).

### 3.3. Associations between Sociodemographics, Social, Dietary, Health Risk Behaviour, Mental Health, Childhood Abuse and Prevalence of Overweight/Obesity

Bivariate analysis among men found that younger age (16–19 years), being from a wealthy family background, coming from a higher income country, lack of organized religious activity, dietary behaviour (trying to eat fiber, and avoiding fat and cholesterol), health risk behaviour (physically inactivity, current tobacco use), poor mental health (depression) and childhood abuse (physical and sexual) were associated with overweight or obesity, while in multivariate regression analysis younger age (16–19 years), coming from a higher income country, dietary behaviour (avoiding fat and cholesterol), health risk behaviour (physically inactive, current tobacco use) and childhood physical abuse were associated with overweight and obesity.

Bivariate analysis among women found that older age (22 or more years), coming from a higher income country, frequent organized religious activity, dietary behaviour (trying to eat fibre, avoiding fat and cholesterol), poor mental health (depression symptoms, PTSD symptoms) and childhood abuse (physical and sexual) were associated with overweight and obesity, while in multivariate regression analysis older age (22 or more years), coming from a higher income country, frequent organized religious activity, dietary behaviour (avoiding fat and cholesterol), poor mental health (PTSD symptoms) and physical childhood abuse were associated with overweight or obesity (see Table 4).

**Table 4:** Associations between BMI overweight/obesity and social, dietary, health behaviours, mental health and abuse in university students from 22 low and middle income and emerging economy countries, 2013.

Variable	Men		Women	
	UOR (95% CI)	AOR (95% CI)	UOR (95% CI)	AOR (95% CI)
**Socio-Demographics**				
*Age in years*				
16–19	1.00	1.00	1.00	1.00
20–21	0.77 (0.67–0.89) ***	0.76 (0.64–0.90) ***	0.97 (0.87–1.10)	0.90 (0.77–1.05)
22 or more	1.02 (0.90–1.17)	0.94 (0.80–1.11)	1.39 (1.22–1.59) ***	1.38 (1.18–1.60) ***
Wealth				
Wealthy	1.00	1.00	1.00	
Quite well off	0.74 (0.59–0.94) *	0.90 (0.66–1.23)	1.00 (0.76–1.32)	
Not well off	0.66 (0.52–0.83) ***	0.83 (0.61–1.14)	1.11 (0.84–1.47)	---
Poor	0.59 (0.45–0.79) ***	0.72 (0.50–1.03)	1.06 (0.76–1.48)	
Country income classification				
Low income	1.00	1.00	1.00	1.00
Lower middle income	1.22 (1.03–1.46) *	3.53 (2.59–4.81) ***	2.59 (1.95–3.31) ***	3.25 (2.39–4.42) ***
Upper middle income	1.27 (1.06–1.51) **	3.35 (2.46–4.550 ***	2.38 (1.93–2.94) ***	2.99 (2.21–4.05) ***
High income	2.54 (1.95–3.31) ***	6.44 (4.34–9.55) ***	6.30 (4.57–8.68) ***	8.87 (5.92–13.28) ***
**Social variables**				
Organised religious activity				
Low	1.00	1.00	1.00	1.00
Medium	1.01 (0.87–1.17)	1.05 (0.87–1.25)	1.26 (1.09–1.45) ***	1.19 (0.99–1.43)
High	0.83 (0.72–0.96) *	0.86 (0.71–1.03)	1.21 (1.05–1.40) **	1.22 (1.03–1.47) *
Social support				
Low	1.00		1.00	
Medium	1.06 (0.92–1.24)	---	0.94 (0.81–1.10)	---
High	1.06 (0.92–1.23)		0.87 (0.75–1.01)	
**Dietary variables**				
Eats red meat at least once a day	1.02 (0.91–1.14)	---	1.05 (0.94–1.16)	---
Try to eat fiber	1.16 (1.03–1.30) *	1.02 (0.87–1.20)	1.30 (1.19–1.44) ***	1.14 (0.96–1.37)
Avoids fat and cholesterol	1.33 (1.18–1.49) ***	1.29 (1.11–1.51) ***	1.38 (1.25–1.52) ***	1.28 (1.12–1.46) ***
Fruit and vegetables (<5 times/day)	0.94 (0.81–1.08)	---	0.98 (0.86–1.11)	---
Skipping breakfast	0.96 (0.86–1.07)	---	1.07 (0.97–1.19)	---
Number of meals a day	1.05 (0.98–1.13)	---	0.95 (0.89–1.02)	---
Number of in-between snacks	1.03 (0.97–1.10)	---	1.02 (0.96–1.09)	---
**Health risk behaviour**	UOR (95% CI)	AOR (95% CI)	UOR (95% CI)	AOR (95% CI)
Physical activity				
Low	1.00	1.00	1.00	
Moderate	0.88 (0.76–1.03)	0.92 (0.77–1.11)	0.88 (0.77–1.01)	---
High	0.82 (0.72–0.93) **	0.78 (0.67–0.91) ***	1.02 (0.90–1.16)	
Current tobacco use	1.33 (1.16–1.51) ***	1.22 (1.03–1.45) *	1.02 (0.82–1.27)	---
Binge drinking (past month)	1.11 (0.97–1.28)	---	1.08 (0.90–1.28)	---
**Mental health and abuse**				
Depression symptoms (severe)	1.29 (1.10–1.51) **	1.13 (0.93–1.39)	1.26 (1.10–1.45) ***	1.15 (0.96–1.37)
PTSD symptoms	1.12 (0.97–1.29)	---	1.24 (1.09–1.40) ***	1.23 (1.06–1.44) **
Child sexual abuse	1.53 (1.08–2.19) *	1.29 (0.83–2.01)	1.39 (1.05–1.85) **	1.03 (0.71–1.47)
Child physical abuse	1.30 (1.02–1.64) *	1.35 (1.01–1.80) *	1.65 (1.32–2.08) ***	1.72 (1.22–2.30) ***

Notes: *** *p* <0.001; ** *p* < 0.01; * *p* < 0.05; UOR = Unadjusted Odds Ratio; AOR = Adjusted Odds Ratio; CI = Confidence Interval.

In sub-Saharan Africa and in Latin America and the Caribbean, female obesity exceeds male obesity both in the general populations and in this study (except for Colombia) reflecting the prevalence of female obesity in these regions. In contrast, in Asia and North Africa (Egypt and Tunisia), male obesity exceeds female obesity. This likely reflects the significant social disadvantage of women in Asia and North Africa. In this study, male obesity exceeded 10% in Bangladesh, India, Philippines, Egypt and Barbados, while female obesity rates exceeded 10% in South Africa, Jamaica and Barbados. Thus, Barbados was the only country in which both male and female obesity rates exceeded 10%, and with 61% tertiary enrollment, these rates reflect a significant national obesity problem, due to high levels of disposable income, diets rich in calorie dense foods and low levels of physical activity. In several countries, tertiary enrollment is low, and likely to be biased towards upper income groups as compared to countries with high rates of enrollment in tertiary education where these results are more likely to reflect the status of this age group in the wider society. Obesity is also associated with the *per capita* income of the country (Table 5), especially in countries where tertiary enrollment is high, thus a better representation of the general population of all 16 to 30 year olds in the population. The study did find that coming from a higher income country was significantly associated with prevalence of overweight/obesity.

The study found among university students from 22 low and middle income and emerging economy countries a prevalence of overweight or obese that compares with previous studies in transitional societies [5,6,7,8,9,10,11,12,13,14,15,16,17,18,19,20,21,22,23,24,25,26,27,28,29,30]. In agreement with a number of studies mainly from Asia [14,15,22], it was found that male gender was associated with overweight/obesity in those populations.

**Table 5:** Socio-economic profile and obesity prevalence of countries in study.

Country	% Tertiary Educaton/5 year Post Secondary Age Group (2011)	*Per Capita* Income	Infant Mortality Rate/1000 Live Births (2012)	Life Expectancy at Birth (2012) Males	Life Expectancy at Birth (2012) Females	% Obesity Males	% Obesity Females
**All**						5.8	5.0
**Asia**							
Bangladesh	13	1815	33	69	71	16.2	5.3
India	23	3870	44	64	68	11.8	7.7
Kyrgyzstan	41	2360	24	66	73	1.7	0.6
Laos	17	2879	54	64	67	4.6	4.2
Pakistan	8	2741	69	64	66	0.0	0.9
Philippines	28 (2009)	4339	24	65	72	11.2	4.5
Russia	75 (2009)	23,589	9	63	75	3.7	0.7
Singapore	n/a	60,800	2	80	85	5.5	2.3
Thailand	53	9660	11	71	79	8.7	5.5
Turkey	61	18,551	12	72	78	4.3	1.8
**Africa**							
Egypt	29	6614	18	69	74	12.5	8.5
Ivory Coast	8 (2010)	2006	76	52	54	0.8	2.1
Madagascar	4	962	41	62	65	0.3	1.0
Mauritius	36	14,902	13	70	78	2.1	4.4
Namibia	n/a	7442	28	64	69	5.4	6.9
Nigeria	n/a	2620	78	53	55	0.7	3.0
South Africa	n/a	11,021	33	56	62	3.8	15.6
Tunesia	35	9636	14	74	78	5.4	5.0
**Carribbean and Latin America**							
Barbados	61	26,488	17	75	81	13.0	18.7
Jamaica	28	5130	14	72	77	5.7	10.7
Colombia	43	10,436	15	76	83	5.0	5.5
Venezuela	78 (2009)	13,267	13	72	80	5.2	4.4

Note: World Bank School enrollment, tertiary (% gross); Gross enrollment ratio. Tertiary (ISCED 5 and 6). Total is the total enrollment in tertiary education (ISCED 5 and 6), regardless of age, expressed as a percentage of the total population of the five-year age group following on from secondary school leaving. Source [48,49].

We found that among women, high prevalence of organised religious activity was associated with overweight/obesity. As in this study, recent research reported a higher BMI in women who were affiliated with a Christian religion [50]. Although weight loss programmes were run in some churches [51], weight management has rarely been addressed by religious lectures or in group discussions in developing countries [52]. This presents an opportunity for religious-based prevention programmes for youth at risk for obesity especially in urban areas. In this study an unexpected finding was that making a conscious effort to avoid fat and cholesterol and trying to eat fiber were associated with overweight/obesity. Other studies have also found this association between intake of fiber and obesity [28]. It is possible that in this study where intention (assessed by conscious effort) to avoid fats and cholesterol which was self-reported, this did not correspond to the actual eating pattern. On the other hand, students who were overweight or obese might have already adopted healthier eating behaviour in order to lose weight and be more accepted by their peers. Since the study design was cross-sectional, causality between dietary variables and overweight/obesity could not be established. 

The finding in this study that lack of physical activity and tobacco use were associated with overweight/obesity has also been found in a number of previous studies [15,17,23,28] In this study, male students had a higher mean frequency of physical activity than female students, and physical inactivity was related to overweight/obesity among males but not among females. Other researchers did not find a link between physical inactivity and overweight/obesity either for male or female students despite showing that the men are more like likely to engage in physical exercise in their free time [53]. Others studies indicate that the relationship between BMI and physical activity occurs only among men [54]. Despite these differences the link between obesity and a sedentary lifestyle has been established.

We found associations between overweight/obesity and poor mental health (PTSD) among women and childhood physical abuse among both men and women. The relationship between overweight/obesity and mental health and childhood abuse have been confirmed in previous studies [55,56]. “*These results demonstrate the long-term impact of childhood physical abuse on weight into adulthood and suggest that physically abused children may be at risk for other adverse health outcomes associated with increased weight. Health professionals need to understand this risk for physically abused children and researchers should identify and evaluate strategies for effective interventions*.” [56].

#### Study Limitations

This study had several limitations. The investigation was carried out with students from one university in each country, mostly in urban centers and inclusion of other centres could have resulted in different results. University students are not representative of young adults in general, especially so in countries where they are a small proportion of the university age population, and the overweight or obesity prevalence and its associated factors may be different in other sectors of the population. The study was a cross-sectional study and the temporal relationships between health behaviour practices and social and health status cannot be established in such studies. Further prospective studies are warranted to understand whether social and health behaviours lead to overweight or obesity or *vice versa*. Apart from anthropometric measurements, a limitation of the study was that all the other information collected in the study was based on self-reporting. It is possible that certain behaviours were under or over reported. Further, several concepts were only assessed with one item such as child abuse, some dietary items and religious involvement. Future studies should include full scales for each concept to be assessed. Finally, certain concepts identified in other studies to be related to overweight/obesity, such as body fat percentage, dietary history [14], family history of risk factors [24] should be included in future studies. 

## 4. Conclusions

The study found a high prevalence of overweight/obesity among university students. Several gender specific health risk practices were identified that can be utilized in health promotion programmes. Universities need to address their obesogenic environment and the need for the university administration to promote healthy life styles as proposed by the WHO Global Strategy on Diet, Physical Activity and Health. Student themselves should be engaged in this process since they also need to be involved in promoting and living healthy lives.
